# Different Biological Pathways Between Good and Poor Inhaled Corticosteroid Responses in Asthma

**DOI:** 10.3389/fmed.2021.652824

**Published:** 2021-03-18

**Authors:** Byung-Keun Kim, Hyun-Seung Lee, Suh-Young Lee, Heung-Woo Park

**Affiliations:** ^1^Department of Internal Medicine, Korea University College of Medicine, Seoul, South Korea; ^2^Institute of Allergy and Clinical Immunology, Seoul National University Medical Research Center, Seoul, South Korea; ^3^Department of Internal Medicine, Seoul National University Hospital, Seoul, South Korea; ^4^Department of Internal Medicine, Seoul National University College of Medicine, Seoul, South Korea

**Keywords:** asthma, gene expression, gene regulatory networks, inhaled corticosteroid, transcription factor, pharmacogenomics, blood

## Abstract

Gene regulatory networks address how transcription factors (TFs) and their regulatory roles in gene expression determine the responsiveness to anti-asthma therapy. The purpose of this study was to assess gene regulatory networks of adult patients with asthma who showed good or poor lung function improvements in response to inhaled corticosteroids (ICSs). A total of 47 patients with asthma were recruited and classified as good responders (GRs) and poor responders (PRs) based on their responses to ICSs. Genome-wide gene expression was measured using peripheral blood mononuclear cells obtained in a stable state. We used Passing Attributes between Networks for Data Assimilations to construct the gene regulatory networks associated with GRs and PRs to ICSs. We identified the top-10 TFs that showed large differences in high-confidence edges between the GR and PR aggregate networks. These top-10 TFs and their differentially-connected genes in the PR and GR aggregate networks were significantly enriched in distinct biological pathways, such as TGF-β signaling, cell cycle, and IL-4 and IL-13 signaling pathways. We identified multiple TFs and related biological pathways influencing ICS responses in asthma. Our results provide potential targets to overcome insensitivity to corticosteroids in patients with asthma.

## Introduction

Blood contains many cells involved in immune responses, which explains why blood cell transcriptomics has been used for the study of asthma, an immune-mediated disease. For instance, it was reported that *MKP-1* and *IL-8* gene expression in peripheral blood mononuclear cells (PBMCs) of patients with asthma was useful in predicting clinical response to corticosteroids (1).

Recent transcriptomic studies have focused on the biological systems that are organized by various molecular entities such as genes, proteins and metabolites as well as the interactions between them. These systems can be visualized as networks, also interchangeably recognized as acyclic graphs, in which components (e.g., genes, proteins, or metabolites) are nodes that are connected by edges (relationships between nodes) (2). One good example is a gene co-expression network based on the similar, or correlated, gene expression patterns (3).

However, correlation does not necessarily imply causation. Gene regulatory networks attempt to identify the influencing patterns of transcription factors (TFs) on gene expression in a mechanistic fashion (4). As reviewed before, the activation or repression of different TFs and their regulatory roles in gene expression may determine the responsiveness to anti-asthma therapy, particularly to anti-inflammatory drugs (5). Qiu et al. found that TFs differentially affected gene expression in lymphoblastoid cell lines from children with asthma that included good and poor responders to inhaled corticosteroid (ICS) treatment by applying gene regulatory networks (6).

The purpose of this study was to assess gene regulatory networks of adult patients with asthma who showed good or poor lung function improvements in response to ICSs. To do this, we began by analyzing genome-wide gene expression levels in PBMCs from adult patients with asthma. Following this, we explored whether gene regulatory networks showed good or poor responder-specific regulatory patterns using the Passing Attributes between Networks for Data Assimilation (PANDA) algorithm. PANDA models information flow through networks under the assumption that both “transmitters” and “receivers” play active roles in modulating regulatory processes (7).

## Materials and Methods

This study was approved by the Institutional Review Board of the corresponding institution (H-1408-051-601 and 2019AN0240) and informed consent was obtained from all study participants.

### Study Populations

**Figure 1:**
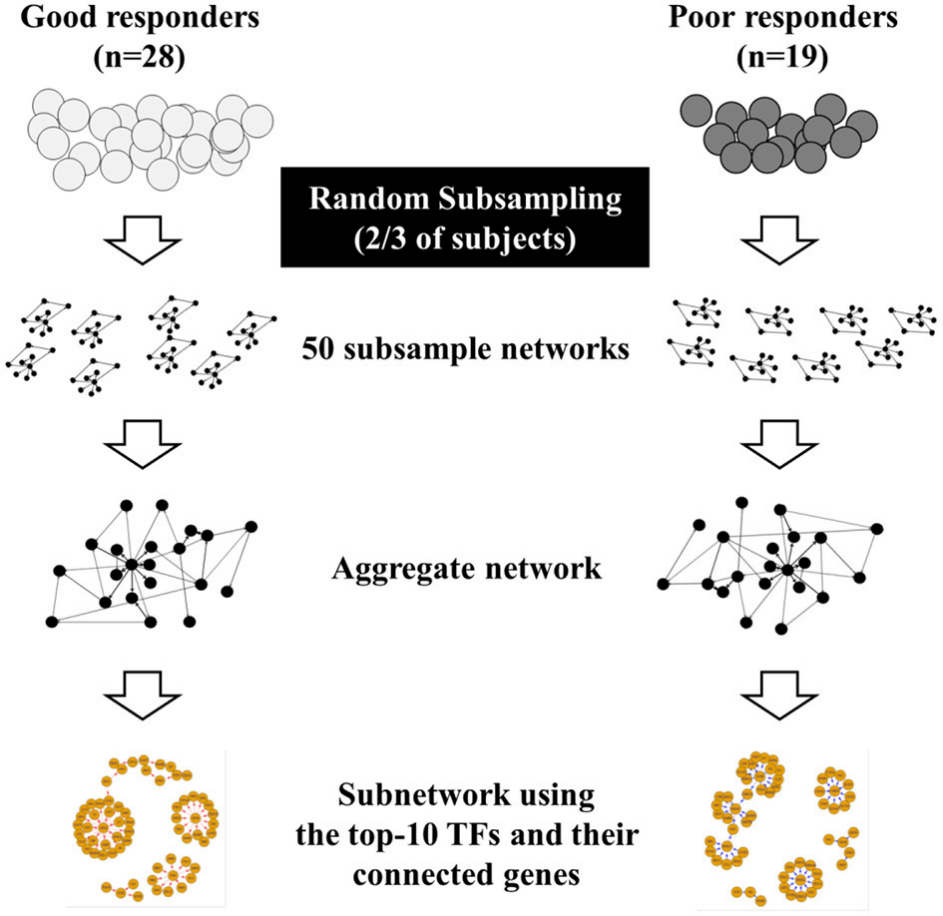
Overall study design. Poor responders (PRs) or good responder (GRs) to inhaled corticosteroids was defined as patients who had less or more than 12% improvement in FEV1 compared to baseline values at 4 weeks after initiation of treatment, respectively, and genome-wide gene expression profiles were obtained from peripheral blood mononuclear cells of participants. Due to the small sample size, two-thirds of the participants were chosen from each GR and PR group at random (without replacement) to form subsamples. We constructed 50 gene regulatory networks in GRs and PRs using PANDA with these 50 subsamples. We then generated a single, aggregate gene regulatory network by averaging Z-scores of edges across the 50 networks identified from subsamples. Finally, we illustrated subnetworks using the top-10 transcription factors (TFs) identified and their differentially-connected genes in each aggregate GR and PR network.

We retrospectively reviewed the medical records of two institutes (Seoul National University Hospital and Korea University Anam Hospital, Seoul, Republic of Korea) and selected adult patients with asthma eligible for our study. The diagnosis of asthma was confirmed when forced expiratory volume in 1 s (FEV1) showed more than 12% (and 200 mL) increase after initiation of treatment. After diagnosis, all of patients were treated with medium dose ICSs (8) and regularly followed up; the pulmonary function measurement was performed every 4 weeks. Current or former smokers were excluded. We explained our study to eligible patients with asthma identified from medical records and enrolled them if they agreed to participate. We defined poor responders (PRs) or good responder (GRs) to ICSs, as patients who had less or more than 12% improvement in FEV1 compared to baseline values at 4 weeks after initiation of treatment, respectively (1). PRs eventually achieved more than 12% improvement in FEV1 responding to ICSs, but it took longer than 4 weeks. The overall study design is presented in Figure 1.

### Gene Expression Arrays

Blood for gene expression analysis was drawn at a stable state, that is, no changes in anti-asthma medications and no acute exacerbations (short-term oral prednisone burst, unexpected clinic visit, and emergency room visit or hospitalization due to asthma symptom aggravation) within 4 weeks prior to blood sampling. PBMCs were isolated and genome-wide gene expression levels were measured using the Affymetrix GeneChip Human Gene 2.0 ST (Affymetrix, Santa Clara, CA, USA). We removed probes with bad chromosome annotations and probes in the X or Y chromosome. We then performed variance-stabilizing transformation and quantile-normalization to reduce technical noises and to make the distribution of expression level for each array closer to a normal distribution.

### Network Analysis

The analysis was performed with R version 4.0.2 (www.r-project.org). We performed PANDA analysis on gene expression profiles from GRs and PRs using the R package “pandaR” (9). network. To seed the PANDA algorithm, we used a mapping between TF motifs and target genes from the TRRUST database (10). This mapping file consists of 8,444 regulatory interactions for 800 TFs and 2,521 target genes. There are 796 TFs in both our gene expression data and the mapping file. These TFs correspond to 9,392 pairs of (TF, gene) and correspond to 2,490 genes in our expression data.

To minimize the effect of outliers in our networks that were built on a smaller sample size, two-thirds of the participants were chosen from each GR and PR group at random (without replacement) to form subsamples. These 50 subsamples were used to construct 50 gene regulatory networks in GRs and PRs. PANDA reports the probability that a connection (edge) exists between a TF and gene in an estimated network as a Z-score (7). We generated a single, aggregate gene regulatory network by averaging Z-scores of edges across the 50 networks identified from subsamples, as described elsewhere (11). We then selected high-confidence edges that had an average edge Z-score >0 in the aggregate GR or PR networks. These edges can be interpreted as edges that are most likely to exist in each aggregate network.

To quantify differences in high-confidence edges, we calculated an edge enrichment score (EES) (11): *EES*_*i*_ = *log*_2_[(*k*^g^_*i*_/*k*^p^_*i*_)/(*N*^g^/*N*^p^)] where *k*^g^_*i*_ and *k*^p^_*i*_ are the (out-degree) number of high-confidence edges for TF *i* in the aggregate GR and PR networks, respectively, and *N*^g^ and *N*^p^ are the total number of high-confidence edges in each network. Note that the EES is positive for edge-enrichment from a particular TF in the aggregate GR network, and negative for edge-enrichment from a particular TF in the aggregate PR network.

### Gene Set Enrichment Analysis

Based on EES, we selected the top-10 TFs from aggregate networks (5 of the highest ones and 5 of the lowest ones). We then identified genes connected to these 10 TFs differentially in the aggregate GR and PR networks by selecting genes whose differences in high-confidence edge Z-scores were >0.75. This means that these genes have at least a 75% chance of existing and being different in each aggregate network. As we assumed that these 10 TFs and their differentially-connected genes were the main drivers in each aggregate network, we constructed GR and PR subnetworks using them. To assign biological meaning to interpretability of each subnetwork, we performed pathway overrepresentation analysis (gene set enrichment analysis) of individual TF and its target genes in the GR and PR subnetworks using “g:Profiler” (database version: e100_eg47_p14_7733820) (12). g:Profiler (https://biit.cs.ut.ee/gprofiler/) provides an adjusted *P*-value calculated in a manner that accounts for the hierarchical relationships among the tested gene sets. g:Profiler utilizes 3 types of biological pathways; KEGG, Reactome, and WikiPathways. As Reactome provides more and diverse signaling pathways including immunological, developmental and kinase, signaling pathways, the drug- or target-based, stress activated, or lipid-mediated signaling pathways compared to the other databases (13), we selected Reactome database.

## Results

**Figure 2:**
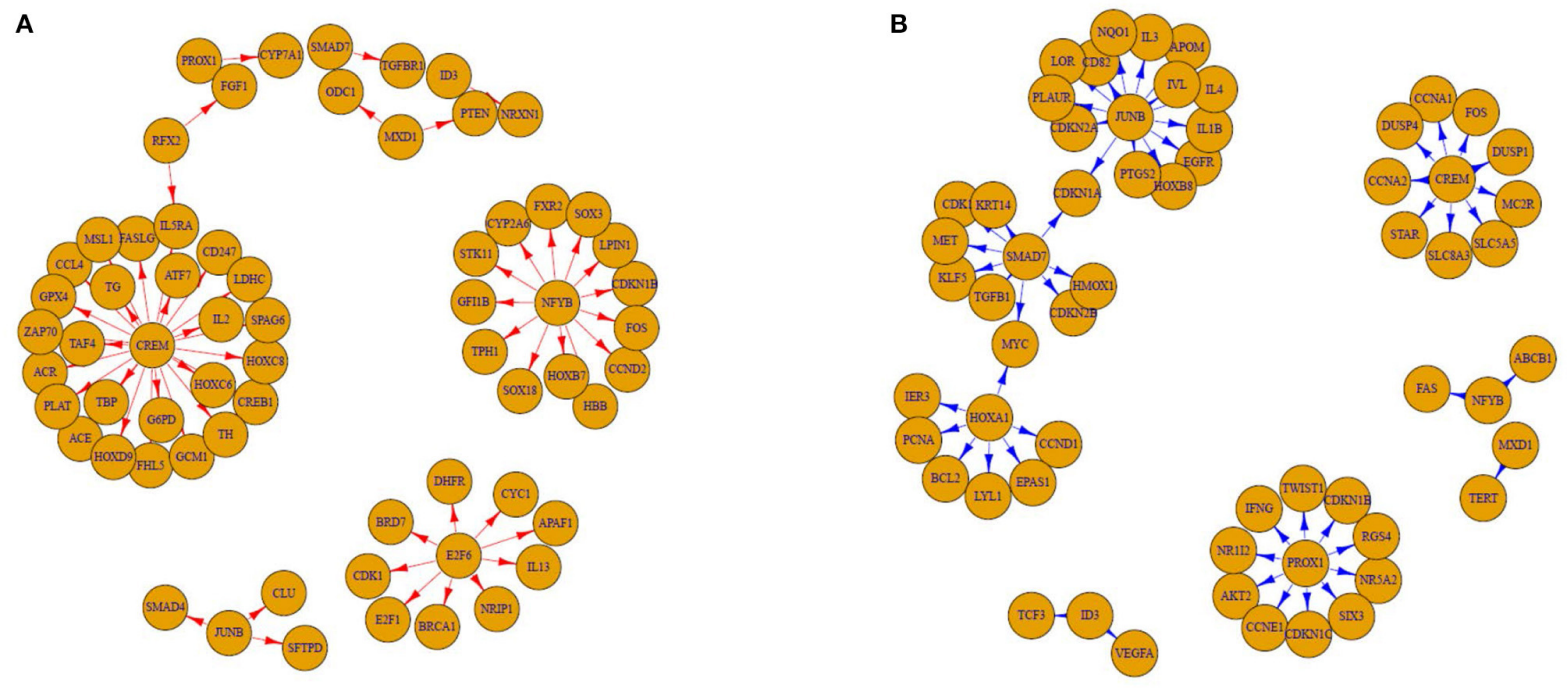
Good (GR) and poor responder (PR) subnetwork made by the top-10 transcription factors and their differentially-connected genes. **(A)** GR and **(B)** PR. Based on edge enrichment score, we selected the top-10 TFs from aggregate networks (5 of the highest ones and 5 of the lowest ones). We then identified genes connected to these 10 TFs differentially in the aggregate GR and PR networks and illustrated subnetworks using them. The edges are directed from TFs to their targeting genes whose differences in high-confidence edge Z-scores are >0.75. This means that these genes have at least a 75% chance of existing and being different in each aggregate network.

A total of 47 adult patients with asthma (28 GRs and 19 PRs) were enrolled. Table 1 summarizes their baseline characteristics. There were no significant differences between GRs and PRs for age, sex, atopy, blood eosinophil counts, or pulmonary functions at baseline (before initiation of treatment). GRs showed significant improvement in FEV1 compared to PRs at 4 weeks after initiation of treatment [694.3 ± 410.9 mL (GR) vs. 78.4 ± 172.5 mL (PR), *P* = 1.82 × 10^−7^), as expected from the definition of 2 groups. Using PANDA, we created aggregate GR and PR networks and identified the top-10 TFs based on EES. Table 2 lists top-5 TFs of those with the highest EES and top-5 TFs of those with the lowest EES. MXD1 has the highest EES, which means that high-confidence edges connecting MXD1 and genes are most greatly enriched in the aggregate GR network. Meanwhile SMAD7 with the lowest EES is positioned in the opposite end. Table 2 also shows absolute numbers of high-confidence edges connecting top-10 TFs and genes in each aggregate GR or PR network and net differences between two aggregate networks. It was difficult to visualize the differential connectivity of TFs and connected genes in the aggregate GR and PR networks, if all TF-gene connections were considered. Hence, we illustrated subnetworks using the top-10 TFs identified and their differentially-connected genes in each aggregate GR and PR network. Figure 2 shows the differential connectivity of top-10 TFs to genes between aggregate GR and PR network. E2F6 and RFX2 are connected to genes with high-confidence in aggregate GR network only, whereas HOXA1 is connected to genes in aggregate PR network only. The other 7 TFs are differentially connected to genes between aggregate GR and PR networks. The name of genes connected to the top-10 TFs in each aggregate network is listed in Table 3. Table 4 summarizes identified biological pathways in which TF and its connected genes in each aggregate network are enriched with adjusted P values less than 0.001. For example, E2F6 and its connected genes in aggregate GR network (APAF1, BRCA1, BRD7, CDK1, CYC1, DHFR, E2F1, IL13, and NRIP1) are significantly enriched in G1/S-Specific Transcription, Transcriptional Regulation by E2F6, G1/S Transition, Mitotic G1 phase and G1/S transition, and Transcriptional Regulation by TP53 pathways. The pathways that were identified helped us understand differences in regulatory control driven by Top-10 TFs between GR and PR. In the GR subnetwork, E2F6, and NFYB supposedly play important roles by regulating cell cycle-related and FOXO-mediated transcription pathways, respectively. Meanwhile, CREM, PROX1, and SMAD7 are crucial in the PR subnetwork controlling cell cycle-related, immune-mediated and TGF-β signaling pathways. Interestingly, JUNB is engaged in both GR and PR subnetworks. However, it differentially regulates biological pathways (TGF-β *vs*. IL-4 and IL-13 signaling pathways). The top-10 TFs identified in this study are not differentially expressed between GR and PR groups (data not shown).

**Table 1:** Characteristics of enrolled patients with asthma.

	**Good responder**	**Poor responder**	***P*-value**
	***n* = 28**	***n* = 19**	
Age (year)	51.9 (13.8)	52.8 (15.6)	0.83
Male	9 (32.1%)	8 (42.1%)	0.75
Atopy	15 (53.6%)	8 (42.1%)	0.64
Blood eosinophil (/μL)	541.7 (220.4)	605.3 (349.6)	0.48
FEV1 (ml)	1,897.1 (501.3)	2,323.1 (987.6)	0.057
FEV1 predicted (%)	67.5 (15.2)	71.9 (19.2)	0.092
FVC (mL)	2,770.3 (684.6)	3,178.4 (1,115.1)	0.12
FVC predicted (%)	79.1 (13.5)	86.2 (15.8)	0.11
FEV1/FVC ratio (%)	66.5 (11.5)	71.9 (10.3)	0.11
FEV1 increase (mL)^a^	694.3 (410.9)	78.4 (172.5)	1.82 × 10^−7^
FEV1 increase (%)^a^	45.5 (42.7)	3.7 (8.1)	1.24 × 10^−4^

*FEV1, Forced expiratory volume in 1 second; FVC, Forced vital capacity*.

a Differences between FEV1 measured at baseline and FEV1 measured at 4 weeks after initiation of treatment. Data are presented as “mean (standard deviation)” except for male and atopy which are presented as “number (%).”

**Table 2:** Top-10 transcription factors identified from aggregate networks based on edge enrichment scores.

**TF**	***n*Edge(GR)**	***n*Edge(PR)**	***n*Diff**	**Log_**2**_(EES)**
MXD1	582	185	397	1.653
NFYB	430	142	288	1.598
E2F6	621	212	409	1.551
CREM	828	290	538	1.514
RFX2	590	209	381	1.497
ID3	235	662	−427	−1.494
HOXA1	279	818	−539	−1.552
JUNB	268	823	−555	−1.619
PROX1	205	678	−473	−1.726
SMAD7	57	323	−266	−2.503

*TF, transcription factor; n, number; diff, difference; EES, edge enrichment score; GR, good responder; PR, poor responder*.

**Table 3:** Top-10 transcription factors and their differentially-connected genes in the aggregate good and poor responder network.

**Transcription factor**	**Connected genes**
**Good responder only**	
E2F6	*APAF1; BRCA1; BRD7; CDK1; CYC1; DHFR; E2F1; IL13; NRIP1*
RFX2	*FGF1; IL5RA*
**Poor responder only**	
HOXA1	*BCL2; CCND1; EPAS1; IER3; LYL1; MYC; PCNA*
**Common**	
CREM	[Good responder] *ACE; ACR; ATF7; CCL4; CD247; CREB1; FASLG; FHL5; G6PD; GCM1; GPX4; HOXC6; HOXC8; HOXD9; IL2; IL5RA; LDHC; MSL1; PLAT; SPAG6; TAF4; TBP; TBP; TG; TH; ZAP70*
	[Poor responder] *CCNA1; CCNA2; DUSP1; DUSP4; FOS; MC2R; SLC5A5; SLC8A3; STAR*
ID3	[Good responder] *NRXN1*
	[Poor responder] *TCF3; VEGFA*
JUNB	[Good responder] *CLU; SMAD4; SFTPD*
	[Poor responder] *APOM; CD82; CDKN1A; CDKN2A; EGFR; HOXB8; IL1B; IL3; IL4; IVL; LOR; NQO1; PLAUR; PTGS2*
MXD1	[Good responder] *ODC1; PTEN*
	[Poor responder] *TERT*
NFYB	[Good responder] *CCND2; CDKN1B; CYP2A6; FOS; FXR2; GFI1B; HBB; HOXB7; LPIN1; SOX18; SOX3; STK11; TPH1*
	[Poor responder] *ABCB1; FAS*
PROX1	[Good responder] *CYP7A1*
	[Poor responder] *AKT2; CCNE1; CDKN1B; CDKN1C; IFNG; NR1I2; NR5A2; RGS4; SIX3; TWIST1*
SMAD7	[Good responder] *TGFBR1*
	[Poor responder] *CDK1; CDKN1A; CDKN2B; HMOX1; KLF5; KRT14; MET; MYC; TGFB1*

**Table 4:** Reactome pathways significantly enriched by the top-10 transcription factors and their differentially-connected genes in the good and poor responder subnetworks.

**TF**	**Good responder**		**Poor responder**	
	**Pathway name**	*P*-value^*^	**Pathway name**	*P*-value^*^
E2F6	G1/S-Specific Transcription	2.02E-06	None	
	Transcriptional Regulation by E2F6	4.57E-06		
	G1/S Transition	0.001104		
	Mitotic G1 phase and G1/S transition	0.001794		
	Transcriptional Regulation by TP53	0.002374		
CREM	None		Phosphorylation of proteins involved in the G2/M transition by Cyclin A:Cdc2 complexes	0.000465
			G2 Phase	0.001549
NFYB	FOXO-mediated transcription	0.006694	None	
JUNB	SMAD2/SMAD3:SMAD4 heterotrimer regulates transcription	0.007895	Interleukin-4 and interleukin-13 signaling	0.000039
			Signaling by interleukins	0.002486
			Cytokine signaling in immune system	0.005848
PROX1	None		Mitotic G1 phase and G1/S transition	0.000534
			PTK6 regulates cell cycle	0.001627
			Cyclin E associated events during G1/S transition	0.005497
			Cyclin A:Cdk2-associated events at S phase entry	0.005905
			AKT phosphorylates targets in the cytosol	0.009838
			TP53 regulates transcription of genes involved in G1 cell cycle arrest	0.009838
SMAD7	None		SMAD2/SMAD3:SMAD4 heterotrimer regulates transcription	0.000503
			Mitotic G1 phase and G1/S transition	0.001057
			Transcriptional activity of SMAD2/SMAD3:SMAD4 heterotrimer	0.001335
			TFAP2 (AP-2) family regulates transcription of cell cycle factors	0.001549
			Signaling by TGF-beta receptor complex	0.005696

* *adjusted P-values*.

## Discussion

In this study, we constructed gene regulatory networks associated with good or poor response to ICSs using gene expression profiles of PBMCs from 47 adult patients with asthma. We identified the top-10 TFs that showed large differences in EES in the aggregate GR and PR networks. We also identified subnetworks made by top-10 TFs and their differentially-connected genes in each aggregate GR and PR network. In addition, these TFs and genes were enriched in distinctly different biological pathways in GRs and PRs. Based on our results, we summarize that TGF-β signaling, cell cycle related, and IL-4 and IL-13 signaling pathways are important in determining responses to ICSs in patients with asthma.

Interestingly, the top-10 TFs and their differentially-connected genes in our regulatory networks showed no significant differences in expression between GRs and PRs (data not shown). It is possible that multiple TFs compete for the same binding site of a target gene, but which one primarily regulates that gene is dependent on the drug response phenotype. Gene regulatory networks provide us an opportunity to model biological processes as information flowing between genes and the potential to identify the underlying causes of the drug response that cannot be captured by differential gene expression networks. PANDA constructs networks based on differential connectivity by comparing differential expression of the TFs. Therefore, the same sets of TFs may regulate different sets of downstream genes between GRs and PRs, as we observed with JUNB in our analysis. Our results indicated that responses between good and poor responders to a certain drug does not necessarily emanate from differential gene expression networks but may instead be from regulatory gene expression networks.

SMAD7 had the lowest EES, which suggests that it may play an important role in the aggregate PR network. Binding of TGF-β to its receptor triggers phosphorylation of SMAD2 and SMAD3 and phosphorylated SMAD2/3 proteins heterodimerize with SMAD4 to generate a complex that moves to the nucleus, where it regulates the expression of target genes (canonical TGF-β signaling) (14). This TGF-β-associated SMAD signaling is tightly controlled by SMAD7, another intracellular SMAD protein (15). Thus, SMAD 7 acts as a negative regulator of the canonical TGF-β signaling pathway. Previously, it was reported that TGF-β impairs therapeutic responses to corticosteroids in chronic airway diseases and the non-canonical signaling pathway is important in this process (16, 17). However, these reports were based on experiments using lung epithelial cells. TGF-β regulates pathologic CD4+ T cell responses by directly suppressing T-bet and GATA-3 expression and by downregulating both Th1 and Th2 cell differentiation (18, 19). In addition, TGF-β can promote the induction of regulatory T cells (20). For dendritic cells, TGF-β can downregulate the antigen-presenting function and expression of co-stimulatory molecules *in vitro* (21). Mice over-expressing Smad7 in T cells develop severe intestinal inflammation in various experimental models (22). In this study, we examined gene expression in PBMCs from adult patients with asthma, contrary to previous studies using lung epithelial cells. This would explain why Smad7 influence increases in the aggregate PR network in our study. In addition, we observed that JUNB is confidently connected with SMAD4 in the GR subnetwork (Figure 2A). A previous report showed that JUNB is a critical activator protein component mediating TGF-β signaling in human breast epithelium (23). Taken together, increased TGF-β signaling in blood cells may confer good response to ICSs in asthma. Although it was recently reported that knockdown of SMAD7 with a specific antisense oligonucleotide that restores endogenous TGF-β activity is not effective for patients with steroid-resistant/dependent Crohn disease (24), a SMAD7-targeting approach is worthy of being searched to treat patients with insensitivity to corticosteroids in asthma.

Corticosteroid possesses an anti-inflammatory action and inhibits various inflammatory chemokines and cytokines, including IL-4 and IL-13 (25). Unexpectedly, we found that JUNB and its related genes in the PR subnetwork were significantly enriched in the IL-4 and IL-13 signaling pathway. Moreover, we observed that E2F6 is connected to IL-13 in the GR subnetwork. Bruhn et al. have suggested an inhibitory role for E2F6 in the regulation of IL-13 and allergy based on gene expression analysis of CD4+ T cells (26). As participants in this study were treated with medium dose ICS, it was possible that the amount of corticosteroids was not enough to suppress IL-4 and IL-13 signaling pathway entirely. Another possible explanation is that corticosteroids in their conventional doses are not sufficient to suppress IL-4 and IL-13 signaling pathway completely in some patients. For example, the 1-week course of prednisone treatment did not show significant changes in bronchoalveolar lavage cells expressing IL-4 and IL-13 mRNA in patients with asthma who were recognized as PRs to corticosteroids (27). In this sense, the role of dupilumab, a monoclonal antibody blocking IL-4 and IL-13 signaling pathway by inhibiting IL-4R alpha, is promising for the management of patients with asthma with reduced response to corticosteroids, as reviewed recently (28). An interesting finding is that the cell cycle (G1/S transition) related pathways are significantly enriched in both GR and PR subnetworks. The accurate transition from G1 (Gap 1) phase of the cell cycle to S (Synthesis) phase is crucial for the control of eukaryotic cell proliferation (29). Since long time ago, we have known that dexamethasone induces irreversible G1 arrest and death of a human lymphoid cell line (30). An arrest of cell cycle progression in the G1/S phase also induced apoptosis of human eosinophils from patients with asthma (31). All these together imply a potential role of the G1/S transition in blood cells in response to corticosteroids. We found that E2F6 and its differentially-connected genes in the GR subnetwork and CREM and PROX1 and their differentially-connected genes in the PR subnetwork are enriched in the G1/S transition related pathways. E2F6 functions as a repressor of E2F-dependent transcription during S phase and thus is presumed to be a cell cycle transcriptional repressor (32). Meanwhile, CREM is implicated in the stimulation of cyclin A transcription at G1/S (33). PROX1, has conflicting roles in cell cycle regulation. PROX1 induces cell cycle arrest in liver hepatocellular carcinoma cells (34), but paradoxically increases proliferation in fetal hepatoblasts (35). These findings suggest that PROX1 regulates the cell cycle in a cell-type-dependent manner. Taken together, cell cycle arrest at G1/S may help to avoid steroid resistance. In addition, it was reported that dexamethasone can stimulate the G1/S transition in human airway fibroblasts in asthma, which may result in airway remodeling (36). In a study reported by Goleva E et al. (1), significantly more dexamethasone was required to suppress *in vitro* T cell proliferation in PBMC from steroid resistant than steroid sensitive asthmatics. Taken together, an intrinsic property related with PBMC proliferation in asthmatics may determine the susceptibility for corticosteroid treatment and thus a new approach focused on the G1/S transition is worthy of being investigated to overcome corticosteroid insensitivity.

RFX2 and HOXA1 may also play an important role in determining response to ICSs. Probably, the number of targeted genes with difference in high-confidence edge Z-scores greater than 0.75 between GR and PR groups is too small to be captured as a specific biologic pathway. As gene expression changes over time, the sampling time of PBMCs in this study may not be the exact time representing the whole picture of RFX2- or HOXA1-related mechanisms.

A small number of participants in this study is a potential limitation. To minimize heterogeneity because of this, we constructed regulatory networks using random subsampling of participants and averaged these networks to identify aggregate GR and PR networks. By doing this, we removed the effect of changes in gene expression that are specific to only one individual (outliers) and focus on changes that are most likely a result of corticosteroid responses. We assumed that TFs with greater differences in high-confidence edges were the main drivers in each aggregate network. Moreover, it was difficult to visualize all the differential connectivity of TFs and their connected genes in the aggregate networks. For these reasons, we selected only the top-10 TFs with a quantified method (EES) focusing on the large-scale changes in edge numbers between two aggregate networks. However, it is possible that other TFs and their related genes excluded from our analysis would have their potential roles in determining ICS responses. Despite taking these precautions, we recognize that future studies are needed for the functional validation of our networks.

In addition, the statistical power and sample size should be considered before generalizing our observations. The performance of gene regulatory network inference algorithms with a genome-wide scale depends on the sample size. It is generally considered that the larger the sample size, the better the gene network inference performance. However, there has not been adequate information on determining the sample size for optimal performance of gene regulatory network inference. In one study using a pseudo gene regulatory network with 6 nodes which is generated from gene-gene associations based on the coefficient of intrinsic dependence, the false networks only appears ≤5 times in 100 simulations for the sample size = 25, 50, and 100 (37). In other study based on the real world gene expression data sets, it was reported that the sample size around 64 is sufficient to obtain acceptable performance of the information-theory-based gene regulatory network inference algorithms (38). We cannot directly apply previous observations to ours, as inference algorithms are different with that of PANDA. However, considering previous reports, we may say that the chance of obtaining false positive gene regulatory networks in this study would not be too much.

In conclusion, we have identified gene regulatory networks to elucidate the differences between GRs and PRs to ICSs in patients with asthma. We identified the top-10 TFs showing different connections between GRs and PRs and found that these top-10 TFs and their differentially-connected genes were significantly enriched in distinct biological pathways, such as TGF-β signaling, cell cycle, and IL-4 and IL-13 signaling pathways. TFs and biological pathways that were identified in this study may be potential targets to overcome insensitivity to corticosteroids in patients with asthma.

## Code Availability

Codes generated during the current study are available upon reasonable request.

## Data Availability Statement

Immediately following publication, individual participant data that underlie the results reported in this article will be able to be shared after de-identification with researchers who will provide a methodologically sound proposal. Proposals should be directed to guinea71@snu.ac.kr.

## Ethics Statement

This study was approved by the Institutional Review Board of the corresponding institution (H-1408-051-601 and 2019AN0240) and informed consent was obtained from all study participants. The patients/participants provided their written informed consent to participate in this study.

## Author Contributions

B-KK, H-SL, S-YL, and H-WP were involved in study conception/design. B-KK, S-YL, and H-WP were involved in data acquisition. All authors were involved in data analysis and/or interpretation, involved in writing/critical review of draft versions of this manuscript and all approved the final version for submission for publication.

## Conflict of Interest

The authors declare that the research was conducted in the absence of any commercial or financial relationships that could be construed as a potential conflict of interest.

## Abbreviations

EES: Edge Enrichment Score
FEV1: Forced expiratory volume in 1 second
GR: Good responder
ICS: Inhaled corticosteroid
PANDA: Passing Attributes between Networks for Data Assimilation
PBMCs: Peripheral blood mononuclear cells
PR: Poor responder
TF: Transcription factor.
